# 3,6'‐disinapoyl sucrose attenuates Aβ_1‐42_‐ induced neurotoxicity in *Caenorhabditis elegans* by enhancing antioxidation and regulating autophagy

**DOI:** 10.1111/jcmm.17153

**Published:** 2022-01-19

**Authors:** Xiaoli Tang, Yuming Zhao, Yanan Liu, Yang Liu, Yue Liu, Fenxi Niu, Fang Fang

**Affiliations:** ^1^ School of Chinese Materia Medica Beijing University of Chinese Medicine Beijing China; ^2^ Department of Pharmacology School of Basic Medical Sciences Capital Medical University Beijing China; ^3^ State Key Lab for Structural Chemistry of Unstable and Stable Species Beijing National Laboratory for Molecular Sciences Institute of Chemistry Chinese Academy of Sciences Beijing China

**Keywords:** 3,6'‐disinapoyl sucrose, Alzheimer's disease, autophagy, *Caenorhabditis elegans*, oxidative stress, β‐amyloid

## Abstract

The aggregation of β‐amyloid (Aβ) has the neurotoxicity, which is thought to play critical role in the pathogenesis of Alzheimer's disease (AD). Inhibiting Aβ deposition and neurotoxicity has been considered as an important strategy for AD treatment. 3,6'‐Disinapoyl sucrose (DISS), one of the oligosaccharide esters derived from traditional Chinese medicine Polygalae Radix, possesses antioxidative activity, neuroprotective effect and anti‐depressive activity. This study was to explore whether DISS could attenuate the pathological changes of Aβ_1‐42_ transgenic *Caenorhabditis elegans* (*C*.* elegans*). The results showed that DISS (5 and 50 μM) treatment significantly prolonged the life span, increased the number of egg‐laying, reduced paralysis rate, decreased the levels of lipofuscin and ROS and attenuated Aβ deposition in Aβ_1‐42_ transgenic *C*. *elegans*. Gene analysis showed that DISS could up‐regulate the mRNA expression of *sod*‐*3*, *gst*‐*4*, *daf*‐*16*, *bec*‐*1* and *lgg*‐*1*, while down‐regulate the mRNA expression of *daf*‐*2* and *daf*‐*15* in Aβ_1‐42_ transgenic *C*. *elegans*. These results suggested that DISS has the protective effect against Aβ_1‐42_‐induced pathological damages and prolongs the life span of *C*. *elegans*, which may be related to the reduction of Aβ deposition and neurotoxicity by regulating expression of genes related to antioxidation and autophagy.

## 1 INTRODUCTION

Alzheimer's disease (AD) is a progressive neurodegenerative disease associated with ageing. The main clinical manifestation of AD patients is cognitive dysfunction and memory deterioration. The pathological features in the AD brain are senile plaques composed of β‐amyloid (Aβ) peptide, neurofibrillary tangles composed of hyperphosphorylated tau and neuron loss.^1^, ^2^ Although the increasing number of AD patients and the high cost of treatment bring heavy burden to society and families, there are no effective treatments to cure or reverse the progress of AD except only a few drugs approved by FDA to improve the symptoms.

The pathogenesis of AD is very complex and still not fully understood. Accumulating evidences showed that the misfolding and aggregation of Aβ can induce mitochondrial dysfunction, oxidative stress, neuronal apoptosis, hyperphosphorylated Tau protein and lead to the progressive impairment in memory and cognition, which is involved in the pathogenesis of AD.^3^, ^4^, ^5^ So, reducing Aβ deposition and neurotoxicity is considered as a significant target for anti‐AD drug research and effective clinical strategy for the prevention and treatment of AD.^6^, ^7^

*Caenorhabditis elegans* (*C*. *elegans*) is a free‐living, non‐parasitic nematode, which has been considered as a model organism for the study of ageing and age‐related diseases, including AD and Parkinson's disease. It has many advantages such as small body size, rapid life cycle from egg to adult, short life‐span and high reproducibility, which make it easy for cultivation. The body of *C*. *elegans* is completely transparent and high sensitivity for RNA interference, which facilitates the study of its biology and visualization. Especially, nerve system of *C*. *elegans* is simple, 40% of nematode genes are homologous to humans, and many genes and molecular in *C*. *elegans* have been identified to mediate learning and memory processes. Transgenic *C*. *elegans* that expressed human Aβ_1‐42_ or Tau have been constructed and used for screening anti‐AD drug, identifying the underlying molecular mechanism of the compounds and revealing the pathogenesis of AD.^8^, ^9^, ^10^

Traditional Chinese medicine Polygalae Radix (Yuanzhi, Root of *Polygala tenuifolia* Willd.) has the efficacies of tranquillizing mind and improving the intelligence, which is often used for insomnia and amnesia.^11^ Modern pharmacological studies showed that the extracts of Polygalae Radix could prevent glutamate, Aβ and corticosterone‐induced cell injury, improve dysfunction of learning and memory in multiple AD animal models, mediate depression and exert sedative‐hypnotic effect.^12^, ^13^, ^14^, ^15^, ^16^ 3,6'‐disinapoyl sucrose (DISS) is a kind of oligosaccharide ester derived from Radix Polygala, which has been used as one of the quality control indices of Polygalae Radix in the Chinese Pharmacopoeia (2015 edition).^11^ DISS has antioxidative effects, neuroprotection and anti‐depression in cells and animal model.^17^, ^18^, ^19^, ^20^ DISS can also inhibit potassium cyanide (KCN)‐induced hypoxia in mice.^21^ The sinapoyl moiety in the structure of DISS is an important moiety for cerebral protection and cognitive improvement.^22^ Our previous studies showed that the oligosaccharide esters (containing DISS) of Polygala Radix attenuated oxidative stress and apoptosis in the SH‐SY5Y cell induced by Aβ_25‐35_ and improved the learning and memory dysfunction induced by scopolamine.^23^, ^24^ We observed the effects of DISS on the Aβ_1‐42_ transgenic *C*. *elegans* and preliminarily explored the relative mechanisms in order to further study the anti‐AD effects of DISS,

## 2 MATERIALS AND METHODS

### 2.1 Chemical reagents

3,6'‐Disinapoyl sucrose (DISS, light yellow powder, purity: 99.41%) was purchased from Must Biotechnology Co., Ltd, dissolved in DMSO (Sigma‐Aldrich) at a concentration of 1 mol/L, and diluted to corresponding concentration for the experiments. Thioflavin‐T (No.27974) was obtained from Beyotime Institute of Biotechnology. 2,7‐dichlorofluorescein diacetate (DCFH‐DA) was obtained from Sigma‐Aldrich. Trizol (No. 180410) and SYBR Green Master Mix (No. 1811582) were provided by Sixin Biotechnology Co. Ltd. RNA reverse transcription Kit (No.766300) was purchased from Toyobo Co. Ltd.

### 2.2 *Caenorhabditis elegans* strains culture and synchronization

The wild‐type *C*. *elegans* strain (N2, Bristol type) and Aβ_1‐42_ transgenic *C*. *elegans* strain (CL2006, *dvIs2* [pCL12 (*unc*‐*54*/human Aβ_1‐42_), pRF4] were used in the experiments. All *C*. *elegans* strains and *Escherichia coli* strain OP50 were purchased from the *Caenorhabditis* Genetic Center, University of Minnesota, MN, USA. The live *E*. *coli* was seeded on the solid nematode growth medium (NGM) as the food source for the growth of all *C*. *elegans* strains at 20°C. The N2 and CL2006 nematodes in the spawning period were transferred to the plates with fresh NGM to lay eggs for 8 h and then removed. The remaining nematode eggs in NGM were incubated at 20°C and the synchronized larvae at stage L4 were used for experiments.

### 2.3 Life‐span assay

Life‐span assay was performed at 20°C. Synchronized N2 and CL2006 nematodes were transferred to fresh prepared plates with DISS (5 and 50 μM) or absent, and assessed every 2 days until the last nematode died. The nematode was considered dead if it did not move at all when it was gently touched with the platinum loop on its head, body and tail for three times. Abnormal dead nematodes were removed. The data of three experiments were summarized together for survival analysis.

### 2.4 Paralysis assay

Nematodes were treated with or without DISS (5 and 50 μM) as described above. The number of paralysed nematodes were recorded every 2 days until the last nematode each group became paralysed. The paralysis standard was identified as the nematode did not move or the head moves only when it was gently touched with platinum loop on its tail for three times. The data of three experiments were summarized together for paralysis analysis.

### 2.5 Egg‐laying assay

Egg‐laying assay was performed on individual nematode. Nematodes were treated with or without DISS (5 and 50 μM) as mentioned above. The culture media was changed every 24 h until the end of egg‐laying. The total number of eggs of each nematode was counted.

### 2.6 Analysis of lipofuscin and reactive oxygen species (ROS) accumulation

Synchronized nematodes were treated with or without DISS (5 and 50 μM) as described above for 10 days. Nematodes were collected in an Eppendorf tube containing 50 μl of M9 buffer solution; then some nematodes were anaesthetized with sodium azide (30 μm), while others were incubated with DCFH‐DA for 30 min at 20°C, washed with M9 buffer solution for three times and anaesthetized with sodium azide (30 μm). The fluorescence of nematode was visualized and photographed with laser scanning confocal fluorescence microscope (IX81‐FV1000, Olympus). The fluorescence intensity was quantified using Image J software.

### 2.7 Quantification of Aβ aggregation

Nematodes treated with or without DISS were stained with 1 mM of thioflavin T (ThT) in 50% ethanol for 4 h, washed with M9 buffer solution for three times, anaesthetized with sodium azide, fixed on the agarose‐coated slide and finally sealed under a coverslip. The Aβ aggregation in the head region of the nematode was counted by fluorescence microscope (Nicon‐Eclipse Ti‐E) with the excitation and emission wavelength at 488 nm and at 510 nm, respectively.

### 2.8 Gene expression analysis by Real‐Time PCR

Nematodes treated with or without DISS were washed from plates with M9 buffer solution and collected in Eppendorf tubes. The total RNA of nematode pellet was extracted using Trizol reagent and converted to cDNA with cDNA Reverse Transcription Kit. The cDNA of candidate genes was amplified and quantified with SYBR Green Master Mix in StepOne plus Real‐Time PCR System. The primers of gene were shown in Table 1. The relative expression level of each gene was calculated using the comparative cycle threshold method normalized to the reference gene actin. Relative fold ‐changes for transcripts were calculated using 2^−△△CT^ method. The experiments were conducted three times.

**TABLE 1:** The primer of genes

Gene	Primer
*skn−1*	(Forward) 5′‐AGTGTCGGCGTTCCAGATTTC−3′
	(Reverse) 5′‐GTCGACGAATCTTGCGAATCA−3′
*daf−2*	(Forward) 5′‐GGCCGATGGACGTTATTTTG−3′
	(Reverse) 5′‐TTCCACAGTGAAGAAGCCTGG−3′
*daf−15*	(Forward) 5′‐ACAACAGACAGGACCAGGAG−3′
	(Reverse) 5′‐GCATAACCGACTGCAACCAT−3′
*daf−16*	(Forward) 5′‐TTTCCGTCCCCGAACTCAA−3′
	(Reverse) 5′‐ATTCGCCAACCCATGATGG−3′
*sod−3*	(Forward) 5′‐CACACTCTCCCAGATCTCCC−3′
	(Reverse) 5′‐AATTTCAGCGCTGGTTGGAG−3′
*gst−4*	(Forward) 5′‐GCTGAGCCAATCCGTATCAT−3′
	(Reverse) 5′‐GGCTTCAGCTTTGACCATTC−3′
*lgg−1*	(Forward) 5′‐GCCGAAGGAGACAAGATCCG−3′
	(Reverse) 5′‐GGTCCTGGTAGAGTTGTCCC−3′
*bec−1*	(Forward) 5′‐ACGAGCTTCATTCGCTGGAA−3′
	(Reverse) 5′‐TTCGTGATGTTGTACGCCGA−3′
*actin*	(Forward) 5′‐CTACGAACTTCCTGACGGACAAG−3′
	(Reverse) 5′‐CCGGCGGACTCCATACC−3′

### 2.9 Statistical analysis

Data were shown as the means ± SD, and analysed by SPSS 22.0 and GraphPad Prism 6 software. OriginPro 8.0 was used for data plotting. The mean life span and paralysis ratio were analysed by a log‐rank (Kaplan‐Meier) statistical test. One‐way ANOVA was used for other results. *p* < 0.05 was considered as statistically significant.

## 3 RESULTS

### 3.1 3,6'‐Disinapoyl sucrose prolonged the life span of Aβ1‐42 transgenic *C. elegans* CL2006

**FIGURE 1:**
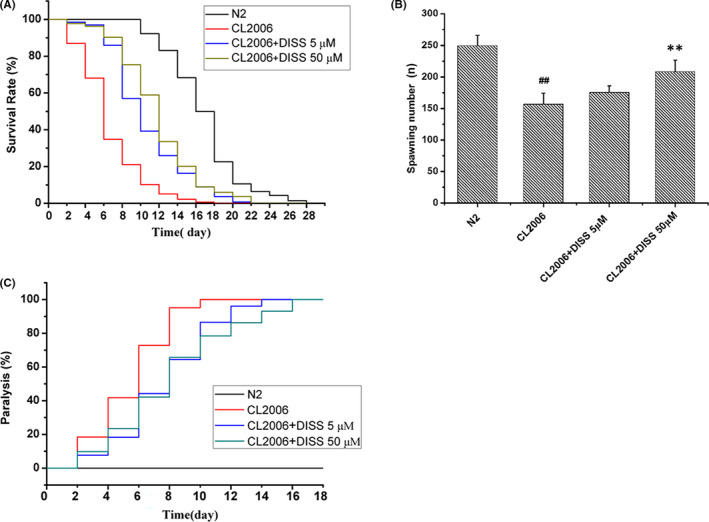
Effects of DISS on life span, egg‐laying and paralysis of Aβ_1‐42_ transgenic *Caenorhabditis elegans* CL2006. (A) The life span of *C*. *elegans*. 200 nematodes in each group were treated with or without DISS, and dead nematodes were counted every 2 days. The curve of life span was made by combining the data of three experiments. (B) The number of egg‐laying of *C*. *elegans*. Data represent means ± SD, the eggs of 6 nematodes each group were counted. ^##^ *p* < 0.01 vs. *C*. *elegans* N2 group; **p < 0.01 vs. Aβ_1‐42_ transgenic *C*. *elegans* CL2006 group. (C) the paralysis rate of *C*. *elegans*. 150 nematodes in each group were incubated with or without DISS, and the paralysed nematodes were recorded every 2 days. The curve of paralysis was made by combining the data of three experiments

The death of N2 nematodes initially appeared on the 10th day and the number of dead nematodes surged to the highest point on the 18th day. The longest life span of N2 nematodes was 28 days. The death of CL2006 nematodes appeared on the 2th day, and the number of dead nematodes surged to the highest point on the 6th day. The longest life span of CL2006 nematodes was 18 days. The median life span of CL2006 nematodes (6.00 ± 0.24) was reduced by about (62.5%) % in comparison with that of N2 nematodes (16.00 ± 0.38). The death peak of CL2006 nematodes treated with DISS (5 μM, 50 μM) appeared on the 8th day and the 10th day, respectively. The longest life span of CL2006 nematodes treated with DISS was 22 days. The median life span of CL2006 nematodes treated with DISS (5 μM, 50 μM) was significantly prolonged compared with that of CL2006 nematodes. The effect of 50 μM of DISS is better than that of 5 μM of DISS (Figure 1A, Table 2).

**TABLE 2:** The effects of DISS on the life span of *Caenorhabditis elegans* (*C*. *elegans*) CL2006. (means ± SD)

Group	Mean (days)	Median (days)
N2	16.68 ± 0.36	16.00 ± 0.38
CL2006	6.58 ± 0.28##	6.00 ± 0.24##
CL2006+DISS 5 μM	10.67 ± 0.35**	10.00 ± 0.47**
CL2006+DISS 50 μM	11.82 ± 0.36**	12.00 ± 0.32**

##*p* < 0.01 vs. *C*. *elegans* N2 group; **p < 0.01 vs. Aβ_1‐42_ transgenic *C*. *elegans* CL2006 group.

### 3.2 3,6'‐Disinapoyl sucrose increased egg‐laying in Aβ_1‐42_ transgenic *C*. *elegans* CL2006

We counted eggs of each nematode in order to investigate whether increased longevity was accompanied by an improvement in fertility. As is shown in Figure 1B, the number of egg‐laying in CL2006 nematodes (156.67 ± 17.63) was reduced by about 37% in comparison to N2 nematodes (249.50 ± 16.49). Compared with CL2006 nematodes, DISS (5 μM, 50 μM) treatment increased the number of egg‐laying of CL2006 nematodes by about 12% and 33%, respectively. The effect of 50 μM of DISS was more obvious (*p* < 0.01).

### 3.3 3,6'‐Disinapoyl sucrose decreased paralysis of Aβ_1‐42_ transgenic *C*. *elegans* CL2006

The Aβ_1‐42_ transgenic *C*. *elegans* CL2006 expresses the deposition of amyloid aggregates in the body wall muscles, which leads to a progressive paralysis in age dependence.^12^, ^16^ As is shown in Figure 1C, N2 nematodes were not paralysed before day 18. All CL2006 nematodes were paralysed on the 10th day, while the DISS (5 μM, 50 μM)‐treated CL2006 nematodes were paralysed on the 14th day and the 16th day, respectively. The paralysis time of 50% CL2006 nematodes was 6.00 ± 0.28 days, while the paralysis time of 50% CL2006 nematodes treated with DISS (5 μM, 50 μM) was 8.00 ± 0.47 and 8.00 ± 0.4 days, respectively.

### 3.4 3,6'‐Disinapoyl sucrose reduced ROS production and lipofuscin accumulation in Aβ_1‐42_ transgenic *C*. *elegans* CL2006

**FIGURE 2:**
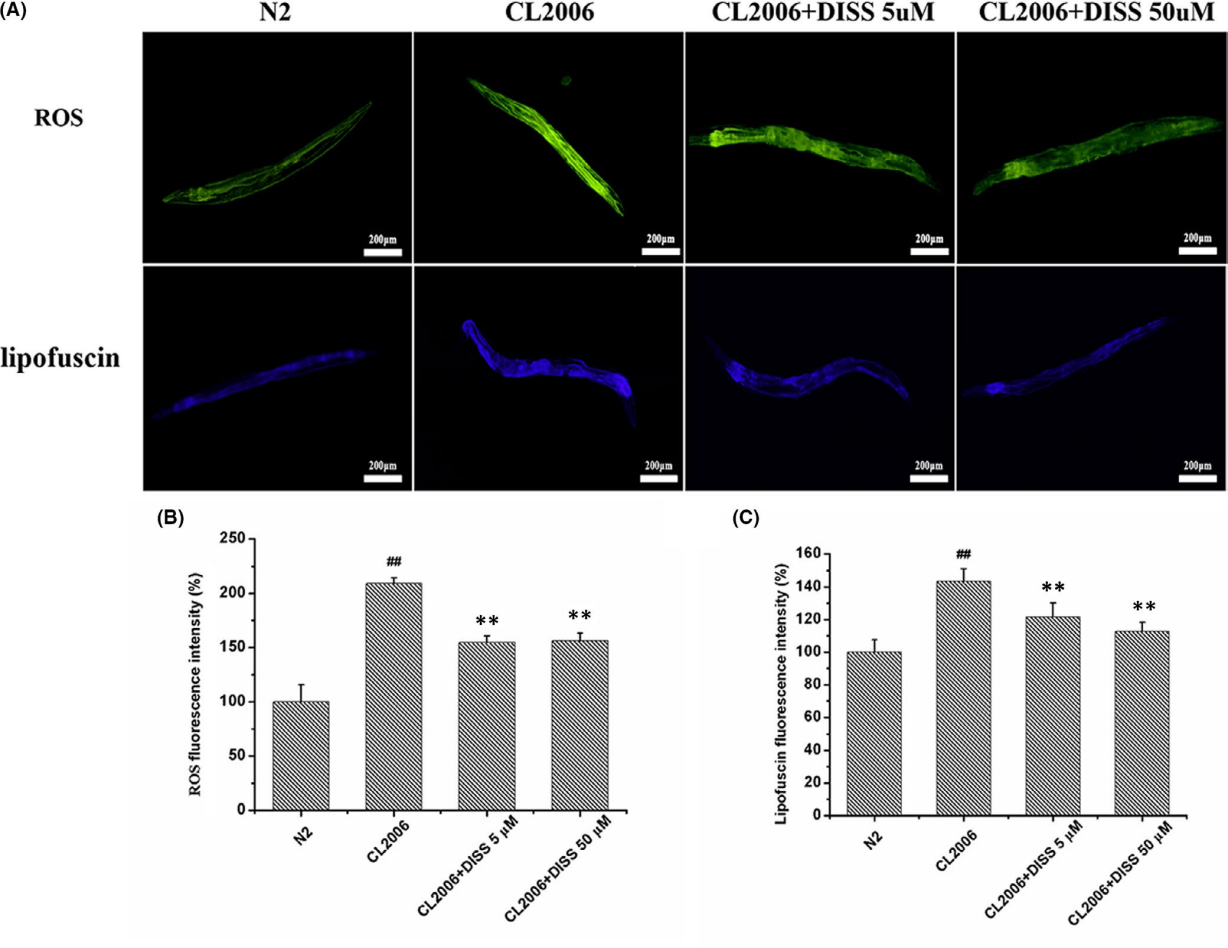
DISS reduced ROS production and lipofuscin accumulation in Aβ_1‐42_ transgenic *Caenorhabditis elegans* CL2006. (A) The representative picture of ROS and lipofuscin. Synchronized nematodes were treated with or without DISS for 10 days. (B and C) Quantitative analysis of the ROS and lipofuscin fluorescence intensity, respectively. Data represent means ± SD, and obtained from 6 nematodes in each group. ^##^ *p* < 0.01 vs. *C*. *elegans* N2 group; *p < 0.05, **p < 0.01 vs. Aβ_1‐42_ transgenic *C*. *elegans* CL2006

The levels of ROS were determined by DCFH‐DA fluorescent probe. Compared with N2 nematodes, the fluorescence intensity was significantly increased in CL2006 nematodes (*p* < 0.01). DISS (5 μM, 50 μM) treatment significantly reduced the fluorescence intensity by about 25%–26% in CL2006 nematodes (*p* < 0.01) (Figure 2). Lipofuscin is a product of oxidized lipid‐protein aggregates, which is deposited in cells. The spontaneous blue fluorescence of lipofuscin was measured by UV irradiation. As is shown in Figure 2, compared with N2 nematodes, the accumulation of lipofuscin in CL2006 nematodes was significantly increased, while DISS treatment (5 μM, 50 μM) significantly reduced the accumulation of lipofuscin in CL2006 nematodes (*p* < 0.01).

### 3.5 3,6'‐Disinapoyl sucrose reduces the content of Aβ in Aβ_1‐42_ transgenic *C*. *elegans* CL2006

**FIGURE 3:**
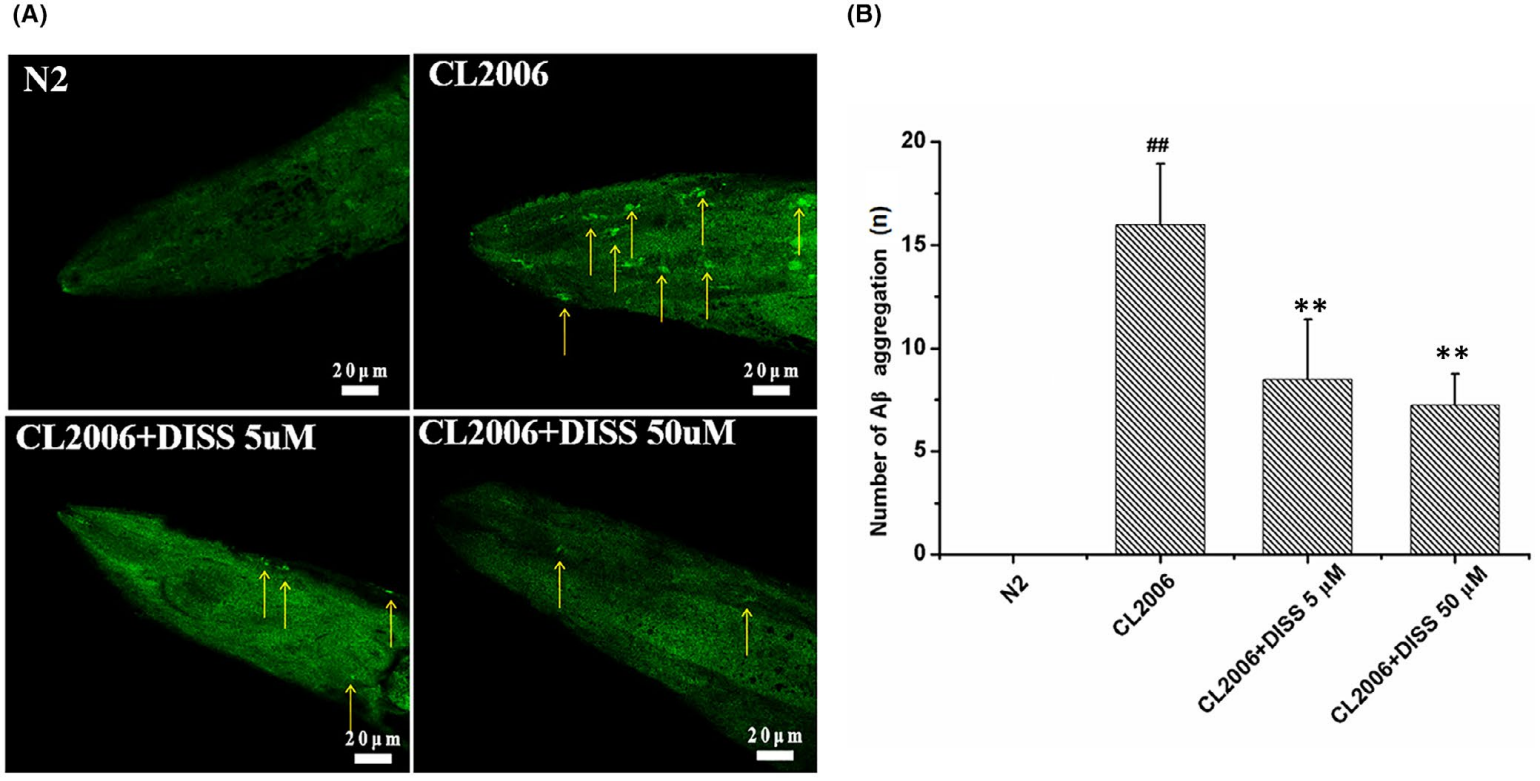
DISS reduced Aβ deposition in Aβ_1‐42_ transgenic *Caenorhabditis elegans* CL2006. (A) The representative picture of Aβ deposition. Synchronized nematodes were treated with or without DISS for 10 days. Yellow arrow represents Aβ deposition. (B) Quantitative analysis of the fluorescence intensity. Data represent means ± SD, and obtained from 6 nematodes in each group. ^##^ *p* < 0.01 vs. *C*. *elegans* N2 group; *p < 0.05, **p < 0.01 vs. Aβ_1‐42_ transgenic *C*. *elegans* CL2006

ThT can bind specifically to Aβ and produce green fluorescence. As is shown in Figure 3, there was no obvious Aβ deposition in the head of N2 nematodes, while a significant increase of Aβ deposition was observed in that of CL2006 nematodes. Compared with CL2006 nematodes, Aβ deposition was decreased in the head of CL2006 nematodes treated with DISS (5 μM, 50 μM).

### 3.6 3,6'‐Disinapoyl sucrose regulated the expression of related gene mRNA in Aβ_1‐42_ transgenic *C*. *elegans* CL2006

**FIGURE 4:**
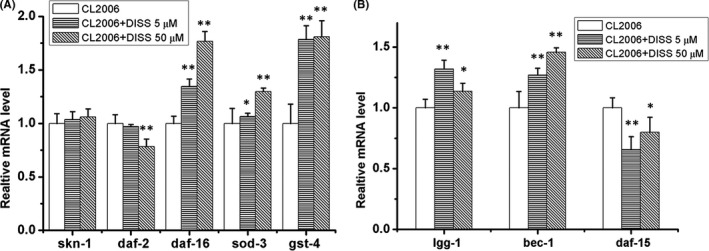
DISS regulated the expression of related gene mRNA in Aβ_1‐42_ transgenic *Caenorhabditis elegans* CL2006. (A) The expression of *skn*‐*1*, sod‐3, gst‐4, *daf*‐*2* and *daf*‐*16 mRNA* in *C*. *elegans* CL2006. (B) The expression of autophagy‐related genes *lgg*‐*1*,*bec*‐*1 and daf*‐*15* mRNA in *C*. *elegans* CL2006. Synchronized nematodes were treated with or without DISS for 10 days. Data represent means ± SD, and obtained from three experiments. *p < 0.05, **p < 0.01. vs. Aβ_1‐42_ transgenic *C*. *elegans* CL2006

The results showed that DISS (5 μM, 50 μM) treatment significantly increased the mRNA expression of *daf*‐*16* gene and antioxidant‐related gene *sod*‐*3* and *gst*‐*4* in CL2006 nematodes, while DISS (50 μM) significantly decreased the mRNA expression of *daf*‐*2* (*p* < 0.01). However, the effects of DISS (5 μM, 50 μM) on the mRNA expression of *skn*‐*1* were not obvious (Figure 4A). In addition, DISS (5 μM, 50 μM) significantly increased the mRNA expression of *lgg*‐*1* and *bec*‐*1* while decreased the expression of TORC1 related receptor gene *daf*‐*15* in CL2006 nematodes (Figure 4B).

## 4 DISUSSION

Previous studies reported that DISS (30, 60 and 100 μM) had neuroprotective effects against glutamate‐induced injury of SH‐SY5Y cells^17^ and DISS (0.4, 10, 50 μg/ml) promoted the proliferation of neural progenitor cells from neonatal rat hippocampus.^25^ According to the concentrations of DISS used in these reports, early experiments in our lab found that the concentrations of DISS (0.5, 5, 50 and 100 μM) had no toxicity to SH‐SY5Y cells and 5 and 50 μM DISS did not affect the life span of *C*. *elegans* N2. So, 5 and 50 μM DISS were selected in this study, and the results showed that DISS could prolong the life span, increase the number of offspring and delay paralysis time of Aβ_1‐42_ transgenic *C*. *elegans* CL2006, indicating that DISS had a protective effect against Aβ‐induced toxicity.

The expression and deposition of Aβ in the body wall muscles are the main characteristics of *C*. *elegans* CL2006. Early research using electron paramagnetic resonance (EPR) spin trapping found that Aβ could generate free radicals in aqueous solution by a metal‐independent, but oxygen‐dependent mechanism, and ROS can be formed as a product of Aβ reactions. Mitochondria are the main organelles for ROS production, and mitochondrial dysfunction has been implicated in the pathogenesis and pathophysiology of AD.^26^ Aβ has the potential to permeate the inner and outer mitochondrial membranes through the translocase of the inner membrane and the translocase of the outer membrane complexes.^27^ Once Aβ is transported into the mitochondrial matrix, Aβ can combine with Aβ‐binding alcohol dehydrogenase or heme group of complex Ⅳ to reduce the function of electron transport chain (ETC), impair complex Ⅳ activity and increase Ca^2+^ efflux and ROS production.^28^, ^29^ Aβ could also block the transport of nuclear‐encoded mitochondrial proteins to mitochondria and cause mitochondrial structural changes, which result in increased mitochondrial fragmentation, decreased mitochondrial fusion, disruption of ETC and synaptic damage.^30^ ROS causes the mitochondrial dysfunction, which further promotes the ROS production and forms a vicious cycle. Especially, ROS damages the protein components, such as lipoprotein receptor‐associated protein, which affects the outflow of Aβ from the brain through the blood‐brain barrier to the blood, leading to the increased deposition of Aβ in the brain.^31^ Therefore, oxidative stress has been considered as an important factor in the pathogenesis and progression of AD,^32^ and a main factor affecting the life span of *C*. *elegans*.^33^ Lipofuscin, a kind of oxidation products of unsaturated fatty acid in organelle fragments, can accumulate in the cells accompanied with the increase of ROS and further aggravate the cell injury.^34^ The accumulation of ROS and lipofuscin was obvious in Aβ transgenic *C*. *elegans* strain CL2006 and GMC101 accompanied with Aβ deposition, and this correlated precisely with the onset of a paralysis phenotype.^28^, ^35^ Oxidative stress is caused by an imbalance between ROS production and antioxidative system in the body. Previous study reported that oligosaccharide ester extracted from *P*. *tenuifolia* roots and DISS had a high antioxidant activity in accelerated senescence‐prone, short‐lived mice; they can increase the superoxide dismutase (SOD) and glutathione peroxidase (GSH‐px) activities in serum and decreased malondialdehyde (MDA) level in liver.^19^ The other study reported that DISS could scavenge DPPH free radicals and ABST free radicals, IC_50_ is 1.08 mg/ml and 0.33 mg/ml, respectively, which indicated that DISS had antioxidative activity.^20^ In this study, we found that DISS could not only significantly reduce the Aβ deposition in the brain, but also decrease the ROS level and lipofuscin accumulation in Aβ_1‐42_ transgenic *C*. *elegans* CL2006, so we speculated that the improvement of DISS against the pathological injury of Aβ_1‐42_ transgenic *C*. *elegans* CL2006 is related to antioxidative activity.

*C. elegans* is the first multicellular animal with a known complete genome sequence, and genes of key molecules related to learning and memory in the brain of human are present in *C*. *elegans* genome, which indicated that *C*. *elegans* model is well suited for studying the molecular, cellular and behaviour of nervous system using whole genome sequence information and morphological analysis. Due to the lack of specific antibodies against *C*. *elegans*, most studies explore the mechanism of drug or pathogenesis of disease at gene level rather than at protein level. In order to explore the molecular mechanism underlying the anti‐oxidative effect of DISS, we further detected the expression of gene related with antioxidation regulation. We found that DISS can increase the expression of antioxidase gene *sod*‐*3* and *gst*‐*4* in Aβ_1‐42_ transgenic *C*. *elegans* CL2006, suggesting that DISS enhanced the function of antioxidase to attenuate oxidative stress. The transcription factor SKN‐1 of *C*. *elegans* is conserved with Nrf2 of mammalian, which controls the antioxidant enzyme expression and maintains protein homeostasis to enhance the body's antioxidant capacity and achieve longevity.^36^ The activation of *skn*‐*1* in *C*. *elegans* attenuates the toxicity of Aβ and extends the life span. However, this protective effect disappears if *skn*‐*1* gene is knocked down by RNAi.^37^ In addition, DAF‐16 is another important transcription factor involved in stress and longevity regulation in *C*. *elegans*, and it is homology with mammalian FOXO protein.^38^, ^39^ *daf*‐*16* RNAi increased the number of paralysed *C*. *elegans*, indicating *daf*‐*16* had the protective effect against the Aβ toxicity.^40^ Activated SKN‐1 and DAF‐16/FOXO can up‐regulate the expression of superoxide dismutase (SOD‐3) or glutathione S‐transferase (GST‐4) to exert antioxidative effects.^41^ DAF‐16 activation is negatively regulated by DAF‐2 in *C*. *elegans*.^42^ Knockdown *daf*‐*2* could significantly attenuate the Aβ‐induced toxicity in *C*. *elegans*, and *daf*‐*2* mutant conferred oxidative stress resistance and increased Mn‐SOD gene expression as well as life‐span extension in *C*. *elegans* by activating *daf*‐*16* gene function.^43^, ^44^ In our study, DISS had no obvious effect on the expression of *skn*‐*1*, while it increased the expression of *daf*‐*16* and decreased the expression of *daf*‐*2* in Aβ transgenic *C*. *elegans* CL2006, indicating that DISS attenuated the Aβ‐induced toxicity and prolonged the life‐span, which may be related to regulating DAF‐2/DAF‐16 mediated antioxidase expression.

Because oxidative stress can promote Aβ production and deposition,^45^, ^46^ it is not difficult to speculate that the decrease of Aβ deposition and neurotoxicity in Aβ transgenic *C*. *elegans* CL2006 treated with DISS may be related to enhancing antioxidative activity through regulating DAF‐2/DAF‐16 pathway. In addition, autophagy dysfunction has been considered to be involved in the pathological alteration of AD, which is also an important factor leading to Aβ deposition due to the inability to remove excessive Aβ.^47^ In addition to the activation of DAF‐16, autophagy is regulated by the mammalian target of rapamycin (mTOR) pathway.^48^ TOR is found in two distinct complexes: TOR complex 1(TORC1, containing LET‐363/TOR and DAF‐15/ Regulatory associated protein of mTOR) and TOR Complex 2 (TORC2, containing LET363/TOR and RICT‐1/Rapamycin‐insensitive companion of mTOR). The inhibition of TORC1 pathway gene *daf*‐*15* and *ragc*‐*1* could initiate autophagy in *C*. *elegans*.^49^ We found that DISS treatment significantly increased the mRNA expression of autophagy‐related gene *bec*‐*1* and *lgg*‐*1*, and decreased *daf*‐*15* gene expression in Aβ transgenic *C*. *elegans* CL2006, suggesting that DISS can activate autophagy induction through inhibiting TORC1 activation, which may be involved in eliminating Aβ. Zhao et al. reported that the extract of Polygalae Radix could induce autophagy via activation of AMPK and inhibition of mTOR to aid the elimination of Aβ peptide. This provided support for our results because DISS is a main active ingredient of Polygalae Radix.^50^ It has been reported that DAF‐16/FOXO negatively regulated the transcriptional expression of *daf*‐*15*,^51^ and the reduced TORC1 activity could also regulate the activity of DAF‐16/FoxO3 and SKN‐1/Nrf‐2 to exert the antioxidation and extend life span in *C*. *elegans*.^52^ Therefore, we speculated that DISS inhibited the expression of *daf*‐*15* in Aβ_1‐42_ *C*. *elegans* CL2006, not only directly activated autophagy but also promoted DAF‐2/DAF‐6 pathway to reduce Aβ deposition and toxicity.

Among the factors affecting Aβ deposition and toxicity, Aβ aggregation is an important part, and the Aβ oligomers are more toxic to neurons than the mature aggregates. ThT is a sulphur pigment that binds specifically to β‐sheet structure of Aβ protein to emit strong fluorescence, which is used to identify the Aβ aggregation and deposition. In this study, we did not observe whether it can directly inhibit Aβ aggregation process in vitro. However, inhibiting oxidative stress or enhancing autophagy might slow down the Aβ aggregation and deposition. Our present study found that DISS could decrease ROS and lipofuscin level, and inhibit the expression of genes related to oxidation and autophagy, which might contribute to the inhibition of Aβ aggregation and deposition. We will observe the effects of DISS on Aβ aggregation in the future work.

Although the pharmacokinetics of DISS in *C*. *elegans* is not obvious, many studies reported that DISS was absorbed into blood very quick when rats were orally administered DISS, Polygalae Radix or compound containing Polygalae Radix.^53^, ^54^ However, the bioavailability of DISS in rats was not very high and it was eliminated from blood, and brain is also very quick because oligosaccharide esters could be hydrolysed to secondary glycosides or aglycones.^55^ However, the absorption of DISS could be promoted by multiple conditions, such as other ingredients in Polygalae Radix, compatibility with other herbs,^56^ disease state^57^ and processing procedure.^53^ In addition, brain distribution research of DISS is limited. A report mentioned that DISS could distribute into brain (~7 ng/g) when rats were orally administered Jia‐Wei‐Qi‐Fu‐Yin (3.0 g/kg), a new developing prescription for treatment on Alzheimer's Disease.^54^ We think that perhaps DISS itself and its metabolites are involved in neuroprotective effects, for example sinapic acid derived from sinapoyl moiety in the structure of DISS showed the cerebral protection and cognitive impairment.^22^ Therefore, metabolism of DISS in vivo and the pharmacological effects of related metabolites should be further study, which is more helpful to illustrate the mechanism.

**FIGURE 5:**
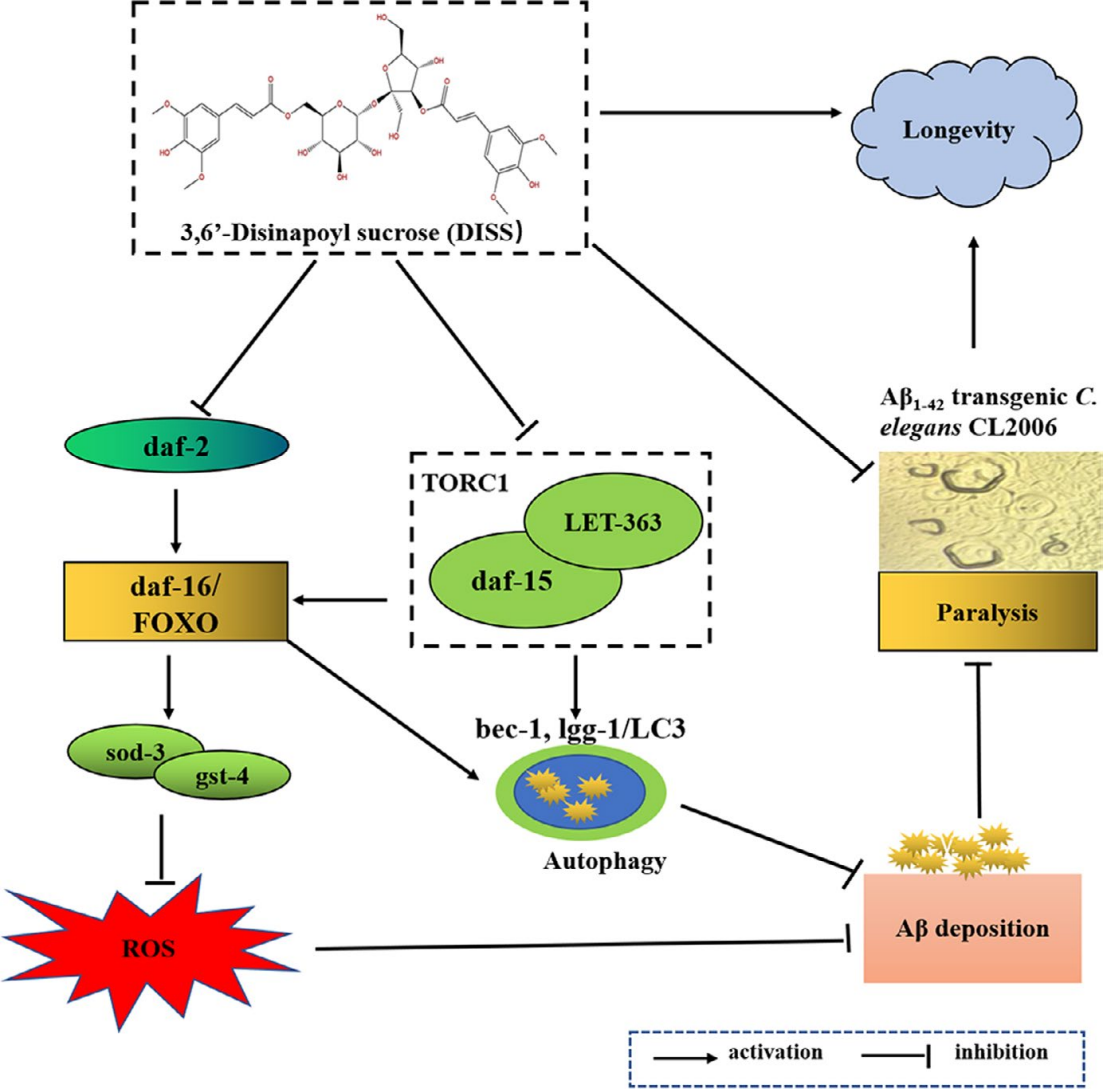
Sketch map of the protection of DISS against pathological alterations in Aβ_1‐42_ transgenic *Caenorhabditis elegans* CL2006

Taking together, the present results (summarized in Figure 5) suggested that DISS could attenuate the Aβ deposition and toxicity in *C*. *elegans* model *of* AD associated with regulating expression of genes related to antioxidation and autophagy, which might be medicated by regulating DAF‐2/DAF‐6 and TORC1 pathway. Although the exact mechanisms of DISS need to be further verified by a variety of experiments, such as using different AD animal models, RNAi and transgenic technology, the present results provided evidences for the material basis research on nootropic effect of Polygalae Radix, which is conducive to promoting the development and utilization of Polygalae Radix. It is worth mentioning that previous studies reported that DISS exerted neuroprotective and antidepressant effects through promoting BNDF expression and enhancing 5‐hydroxytryptamine (5‐HT) and norepinephrine (NE) system.^18^ Recent study found that DISS (60 μM) could strengthen neural stem cells' proliferation, and neuronal differentiation and oral administration of DISS (20 mg/kg) for 4 weeks strikingly rescued the cognitive deficits and hippocampal neurogenesis in adult APP/PS1 transgenic mice.^58^ So, we think that DISS may be a multiple‐mechanism compound, which is worthy of further study as a candidate compound against AD.

## CONFLICT OF INTERESTS

The authors declare that there are no conflicts of interest.

## AUTHOR CONTRIBUTIONS

**Xiaoli Tang:** Data curation (lead); Validation (lead); Writing – original draft (lead). **Yuming Zhao:** Data curation (equal); Methodology (equal); Writing – review & editing (equal). **Yanan Liu:** Visualization (equal). **Yang Liu:** Resources (equal); Software (equal). **Yue Liu:** Supervision (equal). **Fenxi Niu:** Supervision (equal). **Fang Fang:** Methodology (lead); Supervision (lead); Writing – review & editing (equal).

## Data Availability

The data that support the findings of this study are available from the corresponding author upon reasonable request.
